# 

**Published:** 1896-07-04

**Authors:** 


**CADBURY'S COCOA**
"The standard of highest purity at present attainable."—*Lancet.*

**CADBURY'S COCOA**

NO ALKALIES USED.

### THE HOSPITAL

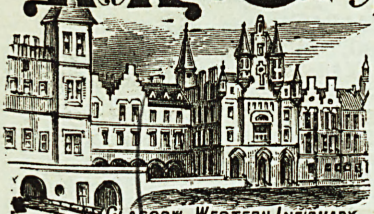

GLASGOW WESTERN INFIRMARY

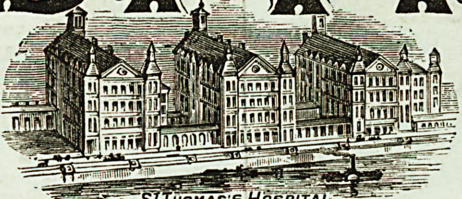

ST. THOMAS'S HOSPITAL

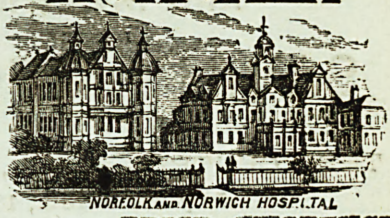

NORFOLK AND NORWICH HOSPITAL

No. 510. Vol. XX.

SATURDAY, JULY 4TH, 1896.

WITH EXTRA NURSING SUPPLEMENT.

[Registered at the General Post Office as a Newspaper for transmission in the United Kingdom and Abroad.]

PRICE TWOPENCE

Per Annum, 10s. 6d., post free.

Monthly Parts, 6d.; post free, 8d.

Ditto per Annum, 8s.6d., post free.

